# The syringe gap: an assessment of sterile syringe need and acquisition among syringe exchange program participants in New York City

**DOI:** 10.1186/1477-7517-6-1

**Published:** 2009-01-12

**Authors:** Daliah I Heller, Denise Paone, Anne Siegler, Adam Karpati

**Affiliations:** 1 New York City Department of Health and Mental Hygiene, New York City, New York, USA

## Abstract

**Background:**

Programmatic data from New York City syringe exchange programs suggest that many clients visit the programs infrequently and take few syringes per transaction, while separate survey data from individuals using these programs indicate that frequent injecting – at least daily – is common. Together, these data suggest a possible "syringe gap" between the number of injections performed by users and the number of syringes they are receiving from programs for those injections.

**Methods:**

We surveyed a convenience sample of 478 injecting drug users in New York City at syringe exchange programs to determine whether program syringe coverage was adequate to support safer injecting practices in this group.

**Results:**

Respondents reported injecting a median of 60 times per month, visiting the syringe exchange program a median of 4 times per month, and obtaining a median of 10 syringes per transaction; more than one in four reported reusing syringes. Fifty-four percent of participants reported receiving fewer syringes than their number of injections per month. Receiving an inadequate number of syringes was more frequently reported by younger and homeless injectors, and by those who reported public injecting in the past month.

**Conclusion:**

To improve syringe coverage and reduce syringe sharing, programs should target younger and homeless drug users, adopt non-restrictive syringe uptake policies, and establish better relationships with law enforcement and homeless services. The potential for safe injecting facilities should be explored, to address the prevalence of public injecting and resolve the 'syringe gap' for injecting drug users.

## Background

In New York City, the authorization and expansion of syringe exchange programs (SEPs) in the 1990s has been associated with significantly reduced HIV prevalence among injecting drug users, from 54% in 1990 to 13% in 2001 [1]. Numerous studies document that, for injecting drug users, adequate syringe access – enough to allow for 1 injection per syringe – is associated with improvements in injection-related risk behaviors and syringe disposal [2-5]. Potential barriers to adequate access or uptake of sterile syringes include: fear and likelihood of police contact [6-10]; and caps on the number of syringes offered by syringe exchange programs [11,12]. Homelessness and injecting in public or semi-public locations increase the likelihood of inadequate syringe coverage and of police contact [9,13-18].

Programmatic data from New York City (NYC) SEPs suggest that many clients visit the programs infrequently and take few syringes per transaction (NYC Department of Health and Mental Hygiene (DOHMH), unpublished data). Separate survey data from individuals using NYC SEP indicate that frequent injecting – at least daily – is common [19]. Together, these data suggest a possible "syringe gap" between the number of injections performed by users and the number of syringes they are receiving from SEP for those injections.

This study was designed to determine the extent to which SEP participants receive adequate numbers of sterile syringes from SEPs relative to their frequency of injection. Also, the study sought to identify reasons why injection drug users do not receive adequate numbers of syringes. Because the study was conceived as an evaluation of syringe exchange programs' effectiveness in meeting the sterile syringe needs of their participants, utilization of alternative sources for sterile syringes, such as pharmacy purchases, was not queried in the study survey.

## Methods

### Study population

This study was conducted among participants of the seven most heavily frequented SEPs among the 13 programs in New York City. During the four-month study period, the mean monthly number of syringes distributed by the seven programs was 153,120. The seven programs provided 89% of all syringes distributed by NYC SEPs during the study period, conducted 86% of all exchanges, and served 4,600 unique participants, representing 83% of all unique participants served by NYC SEPs during this time.

Many SEPs in New York City operate several program sites, exchanging syringes in discrete program sessions at each site at least weekly. Recruitment for the study took place at 15 program sites operated by the seven SEPs, located in ten neighborhoods in the four boroughs where SEPs are authorized: Bronx (4), Brooklyn (3), Manhattan (6), and Queens (2). Not all program sites of each of the seven SEPs were targeted for recruitment: if SEP staff reported patterns of low participation at a particular program site, the site was excluded from study recruitment. This approach resulted in the exclusion of 7 program sites operated by the selected programs.

### Recruitment

Participants were recruited between May and August 2007. During one full program session at each site, one of three trained interviewers was stationed at or within one block of the SEP site. All clients completing syringe exchanges on that day were directed to the interviewer by an SEP worker or invited to participate by the interviewer herself. Clients were offered a $4 public transit card as an incentive to participate. Of the 531 clients who were approached, 504 (95%) agreed to participate. This sample represented 11% of all unique clients using the seven SEPs where recruitment took place during the study period. All participants provided written informed consent. The study protocol was reviewed by the DOHMH Institutional Review Board (IRB) and determined to be exempt from human subjects review.

### Data collection

Data was collected via face-to-face interviews immediately after recruitment, using a structured questionnaire and closed-ended questions. All survey data was anonymous. Participants were asked about demographic characteristics and their frequency of monthly visits to the SEP. Respondents were also queried on the number of syringes they received during the transaction immediately preceding the interview, the number of syringes intended for personal use, how many times on average they used a single syringe, and their frequency of injection, which was recorded however it was reported by the respondent, as a daily, weekly, or monthly rate.

A subset of respondents (N = 135) was also asked a set of open-ended questions regarding the reasons for taking the particular number of syringes they took that day. The subset comprised the first 57 respondents and the first 4 respondents interviewed at each site thereafter.

Of the 504 surveys completed during the study period, 19 were subsequently discarded from the analysis because data measuring the key study variables, syringe need or acquisition, were missing, and an additional 7 surveys were discarded as outliers in the sample (i.e., >500 syringes received today for personal use, and >20 reported daily injections). The final sample included 478 respondents.

### Analysis

Monthly injection frequency was estimated from daily, weekly, or annual reported rates as:

Injections/day * 30, or, Injections/week * 4, or, Injections/year/12.

Participants were classified as having adequate syringe coverage if they reported receiving at least as many syringes from the SEP in the past month as their number of injections during that time. Proportional syringe coverage was calculated as:

Syringes obtained on interview day * Number of visits to SEP in past month/Monthly injecting frequency.

Adequate syringe coverage was defined as a dichotomous variable, with proportionate syringe coverage less than one indicating inadequate coverage.

Logistic regression was used to test associations between SEP participant characteristics and adequate syringe coverage. Predictors significant at the p < 0.05 level in univariate analysis were used in a multivariable regression. Some strata with small numbers (e.g., "Other" race/ethnicity) were not included in the regression models. SAS 9.1 was used for the analysis.

In the sample subset (N = 134) responding to the open-ended question regarding the number of syringes they received that day from the syringe exchange program, individuals generally responded with a single-sentence response to the question. Qualitative analysis coded these responses into at least one of eight categories that emerged as common reasons for not taking more syringes from the program that day.

## Results

As shown in Table 1, the sample was racially and ethnically diverse. The median age was 43 years, and respondents were predominantly male (74%). Homelessness was common (50%).

**Table 1:** Participant characteristics

	**Participants**	**Inadequate syringe coverage**	**Percent with inadequate coverage**
**All**	478 (100%)	260	54
**Race/ethnicity**			
Black/African American	114 (24%)	56	49
Latino/Hispanic	242 (51%)	147	61
White/Caucasian	83 (17%)	34	41
			
**Gender**			
Male	356 (74%)	206	58
Female	119 (25%)	53	45
Transgender	4 (1%)	2	50
			
**Age group**			
19–25 years	19 (4%)	15	79
26–35 years	85 (18%)	54	64
36–45 years	169 (35%)	95	56
>45 years	205 (43%)	97	47
			
**Program site**			
Queens	28 (6%)	9	32
Midtown Manhattan	31 (7%)	12	39
Brooklyn	51 (11%)	23	45
Harlem	111 (23%)	53	48
Lower East Side	84 (18%)	50	60
Bronx	174 (36%)	114	66
			
**Currently homeless**			
Yes	237 (50%)	154	65
No	242 (50%)	107	44
			
**Injected in a public place in past month**			
Yes	237 (49%)	158	67
No	242 (51%)	103	43
			
**Stopped by police while traveling to/from SEP in past month**			
Yes	78 (16%)	53	68
No	401 (84%)	208	52

Nearly half (49%) of respondents reported injecting in a public or semi-public location in the past month. Public injecting was reported in all response categories, including a public bathroom, an apartment hallway, a park, a rooftop, a subway station, and a bank machine enclosure. When public injecting was converted to a scale variable from these six categories, it showed good internal reliability (alpha = .75). In the entire sample, 22% of respondents reported injecting in three or more different public locations in the past month.

One-third of respondents reported being stopped by the police on their way to or from the SEP at least once in the past six months, and one in six reported being stopped at least once in the past month. Police confiscation of syringes and/or injecting supplies in the past month was also reported by 17% of respondents. Sixty-two percent of respondents were concerned about being stopped by police while carrying syringes.

Table 2 shows the injecting and SEP use of the respondents. They reported injecting a median of 60 times per month, visiting the SEP a median of 4 times per month, and obtaining a median of 10 syringes per transaction; more than one in four (35%) reported re-using syringes at least once. Fifty-four percent of participants lacked adequate syringe coverage. Half of respondents received enough syringes to cover, at most, only 80% of their monthly injections, and one-quarter received enough syringes to cover, at most, only 30% of their injections in the past month. In contrast, 46% of participants reported receiving as many or more syringes in a month as they needed for their frequency of injection. In this group, most took many more syringes than their monthly injecting needs. The top quartile reported taking over 4 times their monthly injecting needs.

**Table 2:** Injecting and syringe exchange program use

	**Median**	**Range**	**Interquartile range**
All syringes obtained on day of interview	10	1, 1000	10, 20
			
Syringes obtained for personal use on day of interview	10	1, 400	5, 20
			
SEP use in past month	4	1, 60	2, 8
			
Monthly injection frequency	60	0.2, 600	30, 120
			
Injections per syringe (number)	1	1, 10	1, 2
			
Proportionate syringe coverage: those with coverage ≥ 1 (adequate)	2.3	1, 6.3	1.3, 4.4
			
Proportionate syringe coverage: those with coverage < 1 (inadequate)	0.33	0.01, 0.97	0.17, 0.51

Univariate analysis revealed significant differences in the probability of inadequate syringe coverage between strata of all the study population characteristics (Table 1). Respondent age was inversely related to the likelihood of inadequate syringe coverage, although more than half of all respondents 45 years or younger lacked adequate coverage. Inadequate syringe coverage was also associated with being stopped by the police on the way to or from the SEP in the past month.

In the multivariable analysis (Table 3), the following predictors were significantly associated with inadequate syringe coverage: Black and Hispanic race/ethnicity (Adjusted Odds Ratio [aOR] 3.0 and 2.5, respectively), male gender (aOR 1.6), age between 19 and 25 years (aOR 6.3), receiving syringes in two particular communities (Lower East Side [aOR 4.1] and Bronx [aOR 2.8]), public injecting (aOR 1.9), and homelessness (aOR 1.6). Police interaction did not have a significant independent effect on inadequate syringe coverage.

**Table 3:** Inadequate syringe coverage: multivariable logistic regression results*

	**Adjusted Odds Ratio**	**95% Confidence Interval**
**Race/ethnicity**		
Black/African American	3.0	1.5, 6.2
Latino/Hispanic	2.5	1.3, 4.8
White/Caucasian	Ref	
		
**Gender**		
Male	1.6	1.0, 2.6
		
**Age group**		
19–25 years	6.3	1.2, 32.0
26–35 years	1.7	0.9, 3.1
36–45 years	1.4	0.9, 2.2
>45 years	Ref	
		
**Program site**		
Midtown Manhattan	0.7	0.2, 2.8
Brooklyn	1.8	0.6, 5.4
Harlem	1.7	0.6, 4.6
Lower East Side	4.1	1.4, 12.1
Bronx	2.8	1.0, 7.9
Queens	Ref	
		
**Injected in a public place in past month**	1.9	1.2, 3.0
		
**Currently homeless**	1.6	1.0, 2.5

* 436 observations were used in the analysis, after missing data and low-frequency strata were excluded.

Among the sub-sample responding to the qualitative question regarding the reasons for not taking an adequate number of needles (Table 4), more than one-third reported not needing more syringes. Program limits on syringes ranked second among the reasons why respondents did not take enough syringes, and fear of being stopped, harassed, or arrested by police ranked third.

**Table 4:** Reasons for number of syringes acquired from SEP: qualitative analysis (N = 134)

**Reason cited**	**Frequency**	**Proportion**
Don't want or need more syringes	51	33
Program doesn't give more syringes	38	25
Have more syringes at home	21	14
Concerned about police interactions	14	9
Trying to cut down on injecting drug use	12	8
Don't want to keep syringes at home	7	5
Don't want to carry more syringes	6	4
Plan to come back to SEP later	3	2
TOTAL reasons	152	100

## Discussion

We found that a large proportion of SEP participants in NYC do not obtain adequate numbers of syringes from the SEPs to meet their monthly injecting needs. In addition, characteristics of social marginalization and vulnerability – homelessness and public injecting – were associated with inadequate syringe acquisition. For SEP participants with inadequate coverage, most reported "not needing" more syringes, but many also identified program limits and fear of police contact as main reasons for not obtaining adequate syringes at their most recent visit to the SEP.

The demographic characteristics of this sample did not differ significantly from participants using SEP in urban areas across the United States [20]: they were predominantly male, people of color, and concentrated in the age range from 35 to 55 years old. Of note, half of respondents were homeless, a finding which highlights the particular population reached by SEP in NYC, although also common to many urban SEP in the United States [21].

Inadequate syringe coverage was identified for more than half of respondents, and is of particular concern among the youngest injecting drug users accessing SEP, and in relation to public injecting in NYC. Research indicates that both younger injectors and new initiates to injecting are less likely to be infected with Hepatitis C or HIV [22,23]. However, younger injectors may be less likely to use SEP services [24], and injecting health risk factors such as receptive syringe-sharing are often higher in this group [25,26]. Public injecting was markedly prevalent in this sample, and represents a situational-environmental risk for increased unsafe injecting practices and hygiene [9,27-29]. Current homelessness represented a weaker predictor of inadequate syringe coverage in this sample, as it was partially correlated with public injecting, but has been consistently associated with higher injecting risk behaviors among SEP participants in the US [30].

Other researchers have found that policing around SEP and inadequate syringe coverage for SEP participants is associated with injecting drug users' 'fear' of police and experience with recent arrest [6,8,11,31,32]. In NYC, individuals participating in SEP may legally possess both sterile syringes and 'used' syringes, and program participation is confirmed with a valid 'program card.' Despite these protections, many participants reported policing around SEPs as a particular concern, echoing the findings of other studies [9,17,26,33]. However, policing alone was not a significant independent predictor of inadequate syringe coverage for SEP participants, and may have been especially influenced by the predominance of public injecting in this sample.

One limitation of this study is that participants were not asked about other sources of syringes in the survey. In NYC, sterile syringes are also available at pharmacies and from medical providers who are registered with the NYS Expanded Syringe Access Program (ESAP). It is unclear from this survey whether participants were using other sterile syringe sources to resolve the syringe gap they experienced. In a separate study among a sample of injecting drug users in NYC in 2005, those who reported using SEPs to obtain syringes also reported using other syringe sources at the same rate as non-SEP users, including pharmacies, medical providers, and 'the street.' 'Friends and relatives' represented the only source from which non-SEP users were significantly more likely to obtain their syringes in that sample (NYC DOHMH, unpublished data). However, other studies have found that pharmacies represent a growing source of sterile syringes for injecting drug users [34].

Although the survey item used to measure injection frequency in this sample has not been validated, the frequencies reported are comparable to other, recent assessments of injection frequency among NYC SEP users (NYC DOHMH, unpublished data). While self-reports of SEP visits by SEP participants have been found to under-estimate visits in other studies [35], SEP visits reported by respondents in this study were actually slightly higher, on average, than visit frequencies observed in programmatic data from NYC SEPs (NYC DOHMH, unpublished data). Because data was collected anonymously for this study, no attempt was made to link respondent self-reports on this item to actual SEP utilization.

A straightforward interpretation of finding a "syringe gap" for an injecting drug user is that it represents potentially unsafe injecting practice due to syringe re-use. However, given that many SEP participants with inadequate coverage as defined in this study likely used other sources to obtain sterile syringes, the "syringe gap" may be alternatively interpreted as representing a marker of relatively higher risk of syringe sharing. In a representative sample of injecting drug users in NYC, the odds of reported receptive syringe-sharing increased with the number of syringe sources accessed by respondents, associating risk with irregular syringe access [19].

As with all non-randomized samples, the findings are not necessarily generalizable to the experiences of all injecting drug users in NYC. Furthermore, because recruitment was limited to only seven of the 13 SEPs in operation during the interview period, it is possible that findings do not reflect the experience of all SEP participants in NYC. In addition, respondents may have over-estimated their injecting frequency, contributing to an over-estimation of inadequate syringe coverage in this sample. It is also possible that respondents may represent a disproportionate number of 'high-frequency SEP users,' whose characteristics may differ considerably from 'low-frequency users.' However, respondent recruitment happened routinely at the seven SEPs involved in this study over the course of each month, for the three-month study period.

In order to complete the analysis, certain assumptions were made regarding injecting frequency and quantity of syringes received from the SEP. Both measures were standardized from survey data to a monthly projection, limiting the potential for internal variability in the analysis.

## Conclusion

Nonetheless, some participants of SEP in NYC appear to experience an inadequate supply of syringes, potentially compromising their ability to practice safer injecting hygiene and to protect themselves and their social networks from disease acquisition and transmission. Although HIV prevalence among injectors has dropped substantially in NYC since the introduction of SEP, and HIV incidence in this group has been reduced as syringe coverage has been expanded [36], sterile syringe access initiatives must remain vigilant if these reductions are to be sustained.

Substantial reported homelessness and public injecting practices in this sample represented particular risk contexts for increased police stops and inadequate syringe coverage, and suggest particular avenues for future programmatic development. First, SEPs in NYC should establish integrated working relationships with the city's homeless services system, to support improved syringe access for homeless injectors. Integrated programming could take several different forms, including the establishment of overnight shelters for homeless injectors at SEP, or by direct linkage with SEP, and targeted permanent housing initiatives for active injecting drug users.

Second, improvement in the relationship between and interactions among SEP participants and law enforcement is important for resolving the syringe gap experienced by these injecting drug users. Perceptions and experiences of vulnerability to policing encounters are heightened among homeless and public injectors in particular, and could be improved if law enforcement adopted a 'health promotion' role for safer injecting in this population, in spite of the illicit nature of injecting drug use. Such 'trust-building' can include both formal relationship-building between public authorities for shared public health and public safety efforts, and local, informal relationship-building between SEP and local police precincts.

Third, the problem of program limits on syringe access stems from the prohibition on secondary syringe exchange detailed in the New York State program regulations. This restriction was enforced with the introduction of 'caps' on syringe amounts per transaction, and historically, has been a required element of SEP policies and procedures in New York. However, this policy appears to impede the intended effects of SEP by reducing the availability of sterile syringes to injecting drug users accessing this service. The reinforcement of limits on syringe access through SEP has been identified as a barrier to adequate syringe coverage in other studies [5,12]. Instead of the 'cap' policy, public health authorities can support authorized SEPs to provide sufficient syringes by qualitatively engaging injecting drug users regarding individual drug use and injecting patterns at each transaction [37].

Finally, although politically controversial, the potential for establishing safe injecting facilities in NYC should be explored. Public injecting in the past month was reported by half of respondents, and almost one quarter reported public injecting in three or more locations, indicating this practice was relatively common in this sample of SEP clients [38]. Given these findings, safe injecting facilities might resolve the syringe gap by attracting higher-risk injecting drug users [39-41], while reducing the public disorder caused by public injecting and relieving law enforcement of the effort required to address this problem. A research-based pilot safe injecting facility operating in Vancouver, Canada since 2003 has demonstrated decreased syringe-sharing and improved injecting hygiene [42,43], reduced public injecting and improved public order [40,44], and increased uptake of drug treatment services [45]. Locally, models for safe injecting facilities could be explored, such as attaching mini-facilities to existing SEPs, or by enhancing peer-based interventions [46].

The findings of this survey present important data for the continued development of SEPs in NYC. SEPs should enhance services to younger injectors, to maximize sterile syringe access and safer injecting hygiene education [47], while promoting engagement in drug treatment. Furthermore, program 'caps' limiting syringe uptake should be eliminated. To ensure adequate syringe coverage in NYC, future programming for SEPs must resolve structural impediments, including law enforcement relations and restrictive program policies and practices. SEPs should also address the prevalence of public injecting among participants, and its relationship to homelessness, with program innovations. Initiatives targeting these problems are most likely to improve the syringe gap for SEP users in NYC.

## Competing interests

The authors declare that they have no competing interests.

## Authors' contributions

DH designed the study, managed data collection, assisted with data analysis, and wrote the manuscript. DP assisted with data analysis and manuscript development. AS assisted with study design and piloted data collection. AK assisted with study design, conducted data analysis, and assisted with manuscript development.
